# Are there really autonomous “unconscious” goals that drive behavior? An event-control approach to goals and actions

**DOI:** 10.3389/fpsyg.2014.00723

**Published:** 2014-07-11

**Authors:** Narayanan Srinivasan

**Affiliations:** Centre of Behavioural and Cognitive Sciences, University of AllahabadAllahabad, India

**Keywords:** consciousness, unconscious, goals, event-control, control hierarchy, actions

The article by Huang and Bargh (2014) discusses the role played by conscious and unconscious goals in the behavior of a person. The authors argue for the existence of unconscious goals. In addition, they argue that both conscious and unconscious goals have similar effects on behavior. In this commentary, we focus on the implications of an event-control approach to the conscious or unconscious nature of goals and their influence on perception and action.

The notion of goals and actions performed to achieve those goals indicates a close link between goals, actions performed to achieve the desired goals, and consequent perceptual effect. Such perception-action loops operating at multiple levels form the core of the event-control approach that has been proposed to understand consciousness, self and agency (Jordan, 2003; Kumar and Srinivasan, 2012, 2013, 2014). In this approach, the control system is theorized as a nested hierarchical system in which the lower levels are nested within the higher levels of the system. The conscious goals or intentions would operate at the higher level. The levels also differ in terms of the spatiotemporal extent of events on which control is exercised with the higher levels linked to more distal perception-action events and the lower levels linked to more proximal perception-action events. According to the event-control approach, activity at the higher level constrains activity at the lower level and the amount of control achieved given these constraints at the lower level is passed to the higher level further enabling the change in constraints passed again to the lower level. The nature of self depends on the highest level at which the control is achieved.

The event-control approach has potential implications for the principles proposed in the Huang and Bargh (2014) in discussing the manner in which conscious and unconscious goals influence other mental processes and behavior. Even assuming the existence of unconscious goals (the event-control approach can be neutral about that possibility), the event-control approach has a lot to say about the potential ways in which conscious or unconscious goals influence other processes.

While Huang and Bargh (2014) focus on multiple goals (conscious or otherwise) that potentially compete with each other, it misses the fact that the mind is a nested hierarchy with possible goals at each level in the control hierarchy (Jordan, 2003; Hurley, 2008; Kumar and Srinivasan, 2012, 2014). In addition, both perception and actions are hierarchically organized. Each action (or perception) is composed of other actions (or perceptions) resulting in a hierarchical structure.

According to the event-control approach, a specific priming effect might occur because of the goals at the lower levels and processes that are part of the lower level are activated by the typically conscious higher level goal. This interpretation implies that even if the lower level goal is unconscious, there is a priming effect only when it is activated indirectly by a conscious goal making it non-autonomous even if it produces a subliminal effect on behavior. The effect of the unconscious goal would be indirect, at best arguing against the automaticity principle. Given that the control processes at different levels operate at different spatio-temporal scales, the lower level goals would influence proximal behavior (like immediate priming effects) more than the higher level conscious goals. The influence of these lower level goals would be reduced or negated in dual task or high load conditions indicating that the putative unconscious goals would be susceptible to attentional influences elicited by the conscious task being performed.

The event-control approach argues that not only a higher level goal but relevant sub-goals might also be active at a given time. The reconfiguration principle under-specifies what goals or sub-goals are active assuming only one active (conscious?) goal at a time. The event-control approach does agree with the notion that the active (high level conscious) goal would constrain information processing at the lower levels of the hierarchical control system. In addition, as long as the actions themselves do not compete with each other, simultaneous multiple goal achievement is possible.

In addition, it has to be kept in mind that establishing the consciousness or unconsciousness of goals is not trivial and depends on the methods available for measuring consciousness or unconsciousness (Seth et al., 2008). The notion of unconscious effects based on priming have been questioned in the literature (Newell and Shanks, 2014) based on potential problems with the priming methodology (Rouder et al., 2007). While attempts have been made to refine the priming methodology to produce subliminal priming effects reliably (Finkbeiner, 2011), most studies simply report a null effect in a separate classification task performed with the prime stimuli (Marien et al., 2012). Given these difficulties, it is not clear that the unconscious nature of the goals has been rigorously established in empirical studies so far.

In conclusion, we argue that more rigorous demonstration of unconscious goals is necessary. In addition, it is not clear that an unconscious goal autonomously influences behavior unless it is possibly activated by a conscious goal (which is present even in a subliminal priming task). The notion of nested hierarchical control with multiple sub-goals at lower levels is important for understanding the role of conscious or unconscious goals and intentions in performing actions.

## Conflict of interest statement

The author declares that the research was conducted in the absence of any commercial or financial relationships that could be construed as a potential conflict of interest.
